# Statistical Modeling of Some Cancerous Diseases Using the Laplace Transform Approach of Basic Life Testing Issues

**DOI:** 10.1155/2022/8964869

**Published:** 2022-06-07

**Authors:** M. E. Bakr, Abdulhakim A. Al-Babtain, Saima Khan Khosa

**Affiliations:** ^1^Department of Basic Science, Giza Higher Institute for Engineering, Cairo, Egypt; ^2^Department of Statistics and Operation Research, College of Science, King Saud University, P.O. Box 2455, Riyadh 11451, Saudi Arabia; ^3^Department of Mathematics and Statistics University of Saskatchewan, Saskatoon, SK, Canada

## Abstract

The purpose of the nonparametric statistical test used in this study is to compare different treatment options by looking at failure behavior in recorded survival data. Patients' survival times are documented after using the proposed approach. The observed data's behavior was assumed to be based on used better than aged in the moment generating function order (UBA_mgf_) characteristic or a constant failure rate in this study (exponential scenario). Suppose that the survival data is UBA_mgf_, then the treatment or the machine or system in use produces a better or a higher expected total present value than an older machine governed by an exponential survival function; if the data is exponential, the suggested treatment strategy is ineffective (the recommended treatment approach has neither positive or negative effects on the patients). To guarantee that the suggested statistical test is used correctly, the efficiency and critical values are calculated and compared to those of other tests, and the technique is then applied to medical data.

## 1. Introduction

During the past few decades, different classes of life distributions have been introduced in an attempt to model different aspects of aging which has contributed to the development of new highly efficient statistical tests. In addition, the exponential distribution is the most important member of the life distribution classes because it has significant statistical features. Now, we have a dataset that seems to be either UBA_mgf_ or exponential. To support one of the two assumptions, a statistical test is necessary, indicating which claim is right.

The most well-known classifications of life distributions are IFR, IFRA, NBU, NBUC, NBUC_mgf_, NBUCL, NBRUL, NBRU_mgf_, UBA, UBAC, UBAC(2), and UBA_mgf_. Properties and applications of these aging notions can be found, Navarro and Pellerey [1], Bryson and Siddiqui [2], Barlow and Proschan [3], Abu-Youssef and Bakr [4], Mahmoud et al. [5], EL-Sagheer et al. [6], Esary et al. [7], Navarro J. [8], Cao and Wang [9], Fernandez-Ponce et al. [10], Ali [11], Hassan and Said [12], Ahmad [13], Qureshi et al. [14], Yusuf and Qureshi [15], and Abu Youssef et al. [16, 17] investigated the moment generating function for UBA (UBA_mgf_).

The following are the consequences of the most popular classes of life distributions, which include the most of well-known classes like IFR, UBA, UBAC, and UBA_mgf_:
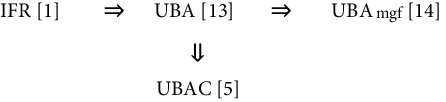


The modeling of lifetime data is used in many applications in reliability theory and biostatistics. The time *T* until some event occurs is the outcome of interest in these applications.

The survivor function is defined as:

(1) $$\begin{array}{c} {\bar{F}}_{t}\left(x\right)=\frac{\bar{F}\left(x+t\right)}{\bar{F}\left(t\right)},\bar{F}\left(t\right)\neq 0. \end{array}$$

*F* has the used better than aged (UBA) property (see Ahmad [13]) if,

(2) $$\begin{array}{c} \bar{F}\left(x+t\right)\geq \bar{F}\left(t\right)e^{-x/\mu \left(\infty \right)},t,x\geq 0,\mu \left(\infty \right)>0, \end{array}$$

**Definition 1 .**

*F* has the moment generating function order of used better than aged (UBA_mgf_) if,

(3) $$\begin{array}{c} \int_{0}^{\infty }e^{sx}\bar{F}\left(x+t\right)dx\geq \frac{\mu \left(\infty \right)}{1-s\mu \left(\infty \right)}\bar{F}\left(t\right),x,t,s\geq 0,0<\mu \left(\infty \right), \end{array}$$

for more details, see Abu Youssef et al. [16].

The primary purpose of this study is to look at how to compare *H*_0_ : *F* is exponential to *H*_1_ : *F* is the largest life distribution UBA_mgf_. The following is the content of the manuscript: using the Laplace transform approach, we give a test statistic for both complete and censored data; for popular alternatives, the Pitman asymptotic efficiency is determined, and selected critical values are listed in Section 2. Finally, in Section 3, we look at several medical science applications to show how important the proposed test is.

## 2. The Statistic Tests

Assume *X*_1_, *X*_2_, ⋯, *X*_*n*_ are random samples from *F*. Here, we create a test statistic to test if *H*_0_ : *F* is exponential, against *H*_1_ : *F* is UBA_mgf_. Nonparametric testing for classes of life distributions has been considered by many authors (see Abu-Youssef et al. [16, 18] and Mahmoud et al. [19].

### 2.1. Complete Data

Using the Laplace approach, the measure of departure (the Laplace methodology is a good generalization of the Goodness of fit technique *β* ≠ 1) can be expressed as:

(4) $$\begin{array}{c} \delta \left(\text{s},\beta \right)=\int_{0}^{\infty }\left[\int_{0}^{\infty }e^{-\beta t}e^{sx}\bar{\text{F}}\left(x+t\right)\text{d}x-\frac{\mu \left(\infty \right)}{1-s\mu \left(\infty \right)}e^{-\beta t}\bar{F}\left(t\right)\right]\text{d}t=\int_{0}^{\infty }\int_{0}^{\infty }e^{-\beta t}e^{sx}\bar{\text{F}}\left(x+t\right)dxdt-\frac{\mu \left(\infty \right)}{1-s\mu \left(\infty \right)}\int_{0}^{\infty }{\text{e}}^{-\beta \text{t}}\bar{\text{F}}\left(\text{t}\right)dt. \end{array}$$

Note that under *H*_0_ : *δ*(s, *β*) = 0 and under *H*_1_ : *δ*(s, *β*) > 0.

The test statistic of the proposed UBA_mgf_ class test is given by the following theorem. Assumed that is the moment generating function exists and finite.

**Theorem 1 .**

Let *X* be the UBA_mgf_ random variable with distribution function *F*; then, based on (4), we have

(5) $$\begin{array}{c} \delta \left(\text{s},\beta \right)=\frac{1}{s\left(\beta +\text{s}\right)}\left(\;\varphi \left(\text{s}\right)-1\right)+\left(\frac{1+\beta +s\left(1-\mu \left(\infty \right)\right)}{\beta \left(\beta +\text{s}\right)\left(1-\text{s}\mu \left(\infty \right)\right)}\right)\left(\;\varphi \left(-\beta \right)-1\right), \end{array}$$

where *φ*(*s*) = ∫_0_^∞^*e*^*sx*^*dF*(*x*).

**Proof**

Starting from (4), we have

(6) $$\begin{array}{c} \delta \left(s,\beta \right)=\int_{0}^{\infty }\int_{0}^{\infty }e^{-\beta t}e^{su}\bar{F}\left(u+t\right)dudt-\frac{\mu \left(\infty \right)}{1-s\mu \left(\infty \right)}\int_{0}^{\infty }e^{-\beta t}\bar{F}\left(t\right)dt,=I-\frac{\mu \left(\infty \right)}{1-s\mu \left(\infty \right)}II, \end{array}$$

where

(7) $$\begin{array}{c} I=\int_{0}^{\infty }\int_{0}^{\infty }e^{-\beta t}e^{su}\bar{F}\left(u+t\right)dudt=\int_{0}^{\infty }\int_{t}^{\infty }e^{-\beta t}e^{s\left(x-t\right)}\bar{F}\left(x\right)dxdt=\frac{1}{\beta +s}\int_{0}^{\infty }e^{st}\left(1-e^{-\left(\beta +s\right)t}\right)\bar{F}\left(t\right)dt=\frac{1}{\beta +s}\left(\frac{1}{s}\left(\varphi \left(s\right)-1\right)-\frac{1}{\beta }\left(1-\varphi \left(-\beta \right)\right)\right), \end{array}$$

(8) $$\begin{array}{c} II=\int_{0}^{\infty }e^{-\beta t}\bar{F}\left(t\right)dt==\frac{1}{\beta }\left(1-\varphi \left(-\beta \right)\right). \end{array}$$

Hence, substituting from (7), (8), to (6), the proof is completed.

(9) $$\begin{array}{c} \delta \left(s,\beta \right)=\frac{1}{\beta +s}\left(\frac{1}{s}\left(\varphi \left(s\right)-1\right)-\frac{1}{\beta }\left(1-\varphi \left(-\beta \right)\right)\right)-\frac{\mu \left(\infty \right)}{1-s\mu \left(\infty \right)}\frac{1}{\beta }\left(1-\varphi \left(-\beta \right)\right)=\frac{1}{s\left(\beta +s\right)}\left(\;\varphi \left(s\right)-1\right)+\left(\frac{1+\beta +s\left(1-\mu \left(\infty \right)\right)}{\beta \left(\beta +s\right)\left(1-s\mu \left(\infty \right)\right)}\right)\left(\;\varphi \left(-\beta \right)-1\right).. \end{array}$$

Without loss of generality, we assume *μ*(∞) is known and equal to one. The empirical estimator of the statistic in (5) can be derived as

(10) $$\begin{array}{c} {\hat{\delta }}_{n}\left(s,\beta \right)=\frac{1}{n\left(\beta +s\right)}\underset{i}{\sum }\left\{\frac{1}{s}\left(e^{{sX}_{i}}-1\right)+\frac{\beta +1}{\beta \left(1-s\right)}\left(e^{-{\beta X}_{i}}-1\right)\right\}, \end{array}$$

and the corresponding invariant test statistic can be obtained as

(11) $$\begin{array}{c} {\hat{∆}}_{n}\left(s,\beta \right)=\frac{{\hat{\delta }}_{n}\left(s,\beta \right)}{\bar{X}}=\frac{1}{n\left(\beta +s\right)\bar{X}}\underset{i}{\sum }\left\{\frac{1}{s}\left(e^{{sX}_{i}}-1\right)+\frac{\beta +1}{\beta \left(1-s\right)}\left(e^{-{\beta X}_{i}}-1\right)\right\}. \end{array}$$

The asymptotic normality of the statistic demonstrated in (5) is presented in the following theorem.

**Theorem 2 . Using *U*-statistics theory (see Lee [20]).**

The statistic *δ*(*s*, *β*) has the features listed below:

As $n⟶\infty ,\sqrt{n}\left({\hat{∆}}_{n}\left(s,\beta \right)-\delta \left(s,\beta \right)\right)$ is asymptotically normal with *μ*_0_ = 0 and *σ*^2^(*s*, *β*), where

(12) $$\begin{array}{c} \sigma^{2}\left(s,\beta \right)=E{\left(\frac{1}{\beta +s}\left\{\frac{1}{s}\left(e^{sx}-1\right)+\frac{\beta +1}{\beta \left(1-s\right)}\left(e^{-\beta x}-1\right)\right\}\right)}^{2}. \end{array}$$

The variance at *H*_0_ is given by:

(13) $$\begin{array}{c} \sigma_{0}^{2}\left(s,\beta \right)=\frac{2}{\left(2\beta +1\right)\left(1-2s\right)\left(\beta -s+1\right){\left(s-1\right)}^{2}}. \end{array}$$

**Proof**

As $n⟶\infty ,\sqrt{n}\left({\hat{∆}}_{n}\left(s,\beta \right)-\delta \left(s,\beta \right)\right)$, then mean *μ*_0_ can be derived by direct calculations, we get

(14) $$\begin{array}{c} \mu_{0}=\int_{0}^{\infty }\left(\frac{1}{\beta +s}\left\{\frac{1}{s}\left(e^{sx}-1\right)+\frac{\beta +1}{\beta \left(1-s\right)}\left(e^{-\beta x}-1\right)\right\}\right)dx=0, \end{array}$$

and the variance

(15) $$\begin{array}{c} \sigma^{2}\left(s,\beta \right)=\mathrm{var}\left(\frac{1}{s\left(\beta +s\right)}\left(\;\varphi \left(s\right)-1\right)+\left(\frac{1+\beta +s\left(1-\mu \left(\infty \right)\right)}{\beta \left(\beta +s\right)\left(1-s\mu \left(\infty \right)\right)}\right)\left(\;\varphi \left(-\beta \right)-1\right)\right),\\ \sigma_{0}^{2}\left(s,\beta \right)=\frac{2}{\left(2\beta +1\right)\left(1-2s\right){\left(s-1\right)}^{2}\left(\beta -s+1\right)}. \end{array}$$

### 2.2. Relative Efficiency

To evaluate the performance of the recommended test technique, we can relate our test to another well-known classes. We employ the test *ξ*(0.01,5) proposed by El-Arishy et al. [21] for the (RNBU_mgf_) class and the test *δ*^∗^(0.01) proposed by Abu-Youssef et al. [16] for the (UBA_mgf_) life distribution. Pitman's relative efficiency asymptotic PARE comparisons are then made using two suggestions in this case:

Linear failure rate family (LFR)

(16) $$\begin{array}{c} {\bar{F}}_{1}\left(x\right)=e^{-x-\left(x^{2}/2\right)\theta },\theta ,x\geq 0. \end{array}$$

Weibull family:

(17) $$\begin{array}{c} {\bar{F}}_{2}\left(x\right)=e^{-x^{\theta }},\theta \geq 1,,x\geq 0. \end{array}$$

Note that *H*_0_ is attained at *θ* = 0 in (i) and *θ* = 1 in (ii). The Pitman's asymptotic efficiency (PAE) of *δ*(*s*, *β*) is equal to

(18) $$\begin{array}{c} \text{PAE}\left(\delta \left(s,\beta \right)\right)=\frac{{\left|\left(\partial /\partial \theta \right)\delta \left(s,\beta \right)\right|}_{\theta ⟶\theta_{0}}}{\sigma_{0}\left(s,\beta \right)}=\frac{1}{\sigma_{0}\left(s,\beta \right)}\left|\frac{-1}{s\left(\beta +s\right)}\int_{0}^{\infty }e^{sx}d{\overset{¯}{F}}_{\theta_{0}}^{\prime }\left(x\right)-\frac{\beta +1}{\beta \left(1-s\right)\left(\beta +s\right)}\int_{0}^{\infty }e^{-\beta x}d{\overset{¯}{F}}_{\theta_{0}}^{\prime }\left(x\right)\right|, \end{array}$$

where ${\overset{¯}{F}}_{\theta_{0}}^{\prime }\left(x\right)=\left(d/d\theta \right){\left.{\overset{¯}{F}}_{\theta }\left(u\right)\right|}_{\theta ⟶\theta_{0}}.$ This leads to:

- PAE in case of the linear failure rate distribution:

(19) $$\begin{array}{c} \text{PAE}\left(\delta \left(0.1,0.2\right)\right)=\frac{1}{\sigma_{0}}\left|\frac{-1}{0.03}\int_{0}^{\infty }e^{0.1x}d\left(-\frac{x^{2}}{2}e^{-x}\right)-\frac{1.2}{0.054}\int_{0}^{\infty }e^{-0.2x}d\left(-\frac{x^{2}}{2}e^{-x}\right)\right|=1.41.\\ \text{PAE}\left(\delta \left(0.4,5\right)\right)=\frac{1}{\sigma_{0}}\left|\frac{-1}{0.002}\int_{0}^{\infty }e^{0.01x}d\left(-\frac{x^{2}}{2}e^{-x}\right)-\frac{1.2}{0.04}\int_{0}^{\infty }e^{-0.2x}d\left(-\frac{x^{2}}{2}e^{-x}\right)\right|=1.401. \end{array}$$

(ii) PAE in case of the Weibull distribution:

(20) $$\begin{array}{c} \text{PAE}\left(\delta \left(0.1,0.2\right)\right)=\frac{1}{\sigma_{0}}\left|\frac{-1}{0.03}\int_{0}^{\infty }e^{0.1x}d\left(-x\ln\left(x\right)e^{-x}\right)-\frac{1.2}{0.054}\int_{0}^{\infty }e^{-0.2x}d\left(-x\ln\left(x\right)e^{-x}\right)\right|=1.01.\\ \text{PAE}\left(\delta \left(0.4,5\right)\right)=\frac{1}{\sigma_{0}}\left|\frac{-1}{0.002}\int_{0}^{\infty }e^{0.01x}d\left(-x\ln\left(x\right)e^{-x}\right)-\frac{1.2}{0.04}\int_{0}^{\infty }e^{-0.2x}d\left(-x\ln\left(x\right)e^{-x}\right)\right|=1.02. \end{array}$$

Direct calculations of PAE of *ξ*(0.01,5), *δ*^∗^(0.01), and our *δ*(0.1, 0.2) and *δ*(0.4,5) are provided in Table 1. The table of efficiencies demonstrates that our statistic test performs well for *F*_1_ and *F*_2_.

In Table 2, we give PAREs of *δ*(0.1, 0.2) and *δ*(0.01, 0.2) with respect to *ξ*(0.01,5) and *δ*^∗^(0.01) whose PAE are mentioned in Table 1.

It is clear from Table 2 that the statistic *δ*(0.1, 0.2) and *δ*(0.4,5) perform well for *F*_1_ and *F*_2_, and it is better than both *ξ*(0.01,5) and *δ*^∗^(0.01) for all cases mentioned above.

### 2.3. Monte Carlo Critical Values

For some selected values *s* and *β* based on 10000 simulated and sample sizes *n* = 10(5)100, we calculate the 90%, 95%, and 99% percentage points of the test statistic of our test ${\hat{\delta }}_{n}\left(\text{s},\beta \right)\;$ in Table 3.

**Figure 1:**
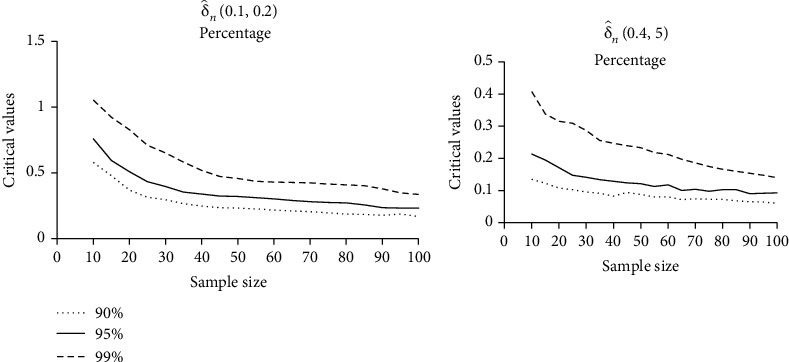
The relation between upper percentile points and sample size of ${\hat{\delta }}_{n}\left(s,\beta \right)$.

The asymptotic normality of our test improves as the critical values decrease and the sample size increases (see Figure 1).

### 2.4. Right Censored Data

One of the most significant developments is due to a unique property of survival data in the life sciences: the data becomes incorrect when certain study participants have not experienced the event of interest at the conclusion of the research or at the time of analysis. Some patients may still be alive or disease-free at the end of the experiment. The subjects' survival time is unknown. This is referred to as censored observations or censored times when individuals are lost to follow-up following a research period.

Using data that has been randomly right censored, the following test statistic is provided to compare *H*_0_ and *H*_1_. Let us write the test statistic such as:

(21) $$\begin{array}{c} \delta_{c}\left(s,\beta \right)=\frac{1}{\left(\beta +s\right)}\overset{n}{\underset{j=1}{\sum }}\overset{j-1}{\underset{k=1}{\prod }}\left(\frac{1}{s}\left(\overset{n}{\underset{m=1}{\sum }}e^{sZ_{\left(m\right)}}-1\left[\overset{m-2}{\underset{p=1}{\prod }}C_{p}^{{Ι}_{p}}-\overset{m-1}{\underset{p=1}{\prod }}C_{p}^{{Ι}_{p}}\right]\right)-\frac{\beta +1}{\beta \left(1-s\right)}\left(1-\overset{n}{\underset{m=1}{\sum }}e^{-\beta Z_{\left(m\right)}}\left[\overset{m-2}{\underset{p=1}{\prod }}C_{p}^{{Ι}_{p}}-\overset{m-1}{\underset{p=1}{\prod }}C_{p}^{{Ι}_{p}}\right]\right)\right). \end{array}$$

We can tabulate the upper percentile points for ${\hat{\delta }}_{\text{c}}\left(s,\beta \right)$ in the same way as before Table 4.

**Figure 2:**
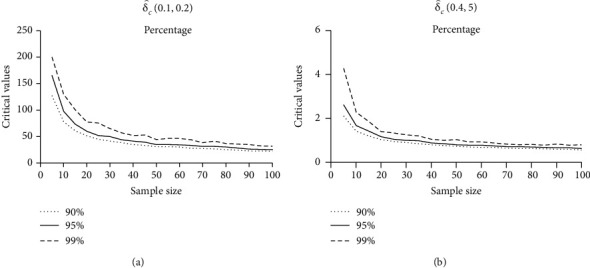
The relation between upper percentile points and sample size of ${\hat{\delta }}_{c}\left(s,\beta \right)$.

The asymptotic normality of our test improves as the critical values decrease and the sample size increases (see Figure 2).

## 3. Applications

To demonstrate the utility of the conclusions in this study, we apply them to certain real-world datasets.

**Example 1 .**

Take a look at the findings of Table 5 Mahmoud et al.; see [19]. The year-ordered values are as follows.

We calculate the statistic ${\hat{\delta }}_{n}\left(0.1,0.2\right)=0.86$ and ${\hat{\delta }}_{n}\left(0.4,5\right)=0.32$, and both values are greater than the critical value of Table 3 in the two cases of ${\hat{\delta }}_{cn}\left(0.1,0.2\right)$ and ${\hat{\delta }}_{n}\left(0.4,5\right)$ as *n* = 40. As a result, we deduce that this dataset is UBA_mgf_ rather than exponential.

**Example 2 .**

The following datasets correspond to 101 patients with advanced acute myelogenous leukemia who were registered with the International Bone Marrow Transplant Registry (Gaitany and Awadhi [22]). 50 of these patients got an allogeneic bone marrow transplant, which rebuilt their immune systems using a pure HLA (histocompatibility leukocyte antigen) class that was similar to the siblings. After receiving significant doses of chemotherapy, 51 patients got an autologous bone marrow transplant, in which their marrow was reinjected to restore a destroyed immune system.

For the 50 allogeneic transplant patients (censored observations), the following are the results of leukemia free-survival times (in months) (see Table 6).

We calculate the statistic ${\hat{\delta }}_{c}\left(0.4,5\right)=1.86$, which is greater than the critical value in Table 4. As a result, we infer that this set of data seems to have the UBA_mgf_ characteristic property rather than the exponential. As a response, the treatment strategy chosen is significant.

For the other 51 autologous transplant patients (censored observations), the following are the results of leukemia free-survival times (in months) (see Table 7).

We calculate the statistic ${\hat{\delta }}_{c}\left(0.4,5\right)=76.06$, which is greater than the critical value in Table 4. As a response, we infer that this set of data seems to have the UBA_mgf_ characteristic property and not exponential.

According to the presented test, the two treatments given to 101 patients have a positive influence (IFR) on their survival lifetime, and so, the results extrapolated from the appropriate sample size can be applied to all patients with advanced acute myelogenous leukemia. However, in this scenario, the proposed test cannot evaluate two distinct treatments because the test results in both cases yielded the same conclusion or choice, with no priority given to selecting the most effective treatment.

**Example 3 .**

In this application, we use the data from Hassan [23] which reflect the ages (in days) of 51 liver cancer patients from the Elminia Cancer Center Ministry of Health Egypt (see Table 8), who entered the medical examination in the year 2000. In the investigation, only 39 patients are seen (right-censored), while the remaining 11 individuals are dropped (missing from the investigation).

Applying the statistic in (14), we calculate ${\hat{\delta }}_{c}\left(0.1,0.2\right)=5.19\times 10^{6}$ and ${\hat{\delta }}_{c}\left(0.4,5\right)=9.06\times 10^{28}$ which both are greater than the critical value of Table 4 in the two cases of ${\hat{\delta }}_{c}\left(0.1,0.2\right)$ and ${\hat{\delta }}_{c}\left(0.4,5\right)$. Then, we conclude that this dataset have UBA_mgf_ property and not exponential. As a result, the treatment strategy chosen is significant.

**Example 4 .**

By analyzing the data reported by Abbas et al. [24], which shows the survival times in weeks of 61 individuals with inoperable lung cancer who were cured with cyclophosphamide, the patients whose therapy was terminated due to a devolving state are represented by 33 uncensored observations and 28 censored observations (see Table 9).

Applying the statistic in (14), we calculate ${\hat{\delta }}_{c}\left(0.1,0.2\right)=528$ and ${\hat{\delta }}_{c}\left(0.4,5\right)=1.2\times 10^{10}$ which both are greater than the critical value of Table 4 in the two cases of ${\hat{\delta }}_{c}\left(0.1,0.2\right)$ and ${\hat{\delta }}_{c}\left(0.4,5\right)$. Then, we conclude that this dataset have UBA_mgf_ property and not exponential.

## 4. Conclusion

In this paper, a statistical test technique has been developed to aid in the quality evaluation of potential treatments for certain cancers. The results of our tests revealed whether the planned treatments had a positive or negative impact on the patients' survival periods. To ensure that the suggested statistical test produces good findings, its efficiency was computed and compared to existing tests. The proposed test can be used to evaluate the efficacy of any treatment approach in any sector of medical research, independent of the type of the treatment method being used. However, as seen in the second application, this test is not advised for comparing two different treatment strategies. But, it is suggested that new nonparametric statistical tests with high efficiency be developed and used to examine the various proposed treatments. It is also suggested that a statistical approach be developed to compare two or more different treatments that cure the same ailment. In addition, the percentage points of the proposed statistics are simulated. The efficacies of our developed tests are compared to El-Arishy et al. [17] and Abu-Youssef et al. [14] based on Pitman asymptotic relative efficiency using some well-known life distributions, namely, linear failure rate family (LFR) and Weibull family. Finally, the findings of the paper are applied to some medical real datasets.

## Figures and Tables

**Table 1:** PAE of *ξ*(0.01,5), *δ*^∗^(0.01), and *δ*(0.01, 0.2) & *δ*(0.4, 5).

Distribution	*ξ*(0.01,5)	*δ* ^∗^(0.01)	*δ*(0.1, 0.2)	*δ*(0.4, 5)
LFR	0.957	1.3	1.41	1.4
Weibull	0.733	0.969	1.01	1.02

**Table 2:** PARE of *δ*(s, *β*) with respect to *ξ*(0.01,5) and *δ*^∗^(0.01).

Distribution	*e*(*δ*(0.1, 0.2), *ξ*)	*e*(*δ*(0.4,5), *ξ*)	*e*(*δ*(0.1, 0.2), *δ*^∗^)	*e*(*δ*(0.4,5), *δ*^∗^)
LFR	1.47	1.46	1.08	1.07
Weibull	1.38	1.39	1.04	1.05

**Table 3:** The upper percentile points of ${\hat{\delta }}_{n}\left(s,\beta \right)$.

	${\hat{\delta }}_{n}\left(0.1,0.2\right)$			${\hat{\delta }}_{n}\left(0.4,5\right)$		
*n*	90%	95%	99%	90%	95%	99%
10	0.580698	0.759687	1.05433	0.135928	0.213795	0.408465
15	0.479687	0.593628	0.922588	0.123036	0.195852	0.337738
20	0.368159	0.510497	0.829183	0.107751	0.17183	0.315951
25	0.313756	0.433008	0.710547	0.102756	0.147881	0.309676
30	0.297414	0.396515	0.652416	0.09621	0.141571	0.287737
35	0.265572	0.353873	0.581266	0.091847	0.134835	0.255733
40	0.248072	0.338621	0.51924	0.082932	0.129161	0.247065
45	0.235582	0.323443	0.47227	0.094326	0.124469	0.240344
50	0.232373	0.32046	0.45834	0.088195	0.121795	0.23436
55	0.225801	0.312739	0.437835	0.080319	0.113748	0.218722
60	0.216938	0.303363	0.430117	0.080764	0.117606	0.213242
65	0.20962	0.288797	0.427172	0.071928	0.101304	0.198197
70	0.206325	0.282416	0.425205	0.074889	0.104063	0.186504
75	0.19657	0.275887	0.416086	0.073234	0.098717	0.175777
80	0.18705	0.273315	0.4091077	0.073124	0.103674	0.166048
85	0.184602	0.257561	0.403561	0.068204	0.103098	0.160891
90	0.176693	0.235779	0.379767	0.06624	0.090171	0.153907
95	0.187	0.232307	0.347403	0.064317	0.092631	0.148411
100	0.16911	0.231752	0.335324	0.060809	0.093562	0.140649

**Table 4:** The upper percentile points of ${\hat{\delta }}_{c}\left(s,\beta \right)$.

	${\hat{\delta }}_{c}\left(0.1,0.2\right)$			${\hat{\delta }}_{c}\left(0.4,5\right)$		
*n*	90%	95%	99%	90%	95%	99%
5	127.2	165.5	200	2.10825	2.61735	4.26729
10	77.9	97.7	129.6	1.42275	1.67823	2.28296
15	61.0	73.3	100.7	1.20137	1.41178	1.86127
20	51.3	59.7	77.7	1.03342	1.16357	1.40993
25	44.6	52.0	75.4	0.947763	1.04889	1.3386
30	41.4	49.9	65.0	0.890431	0.994642	1.24712
35	38.5	43.8	56.9	0.846647	0.978381	1.2031
40	35.0	41.2	52.0	0.794412	0.878939	1.04313
45	33.4	39.3	53.0	0.764744	0.845721	0.996
50	30.6	35.0	44.5	0.727007	0.797613	1.02486
55	30.6	36.0	46.6	0.692838	0.779898	0.939281
60	30.2	34.4	46.3	0.680368	0.783843	0.937925
65	27.7	33.5	43.7	0.677745	0.754965	0.885337
70	27.1	31.2	38.2	0.649531	0.709978	0.827454
75	26.8	31.1	41.5	0.634067	0.710708	0.797883
80	25.3	30.2	36.7	0.634596	0.704642	0.814818
85	24.1	28.5	35.9	0.608614	0.683274	0.773045
90	22.9	26.2	34.9	0.590324	0.667697	0.826769
95	22.7	25.8	32.5	0.591555	0.659182	0.785755
100	22.3	25.2	32.0	0.570232	0.637941	0.791201

**Table 5:** 

0.315	0.496	0.616	1.145	1.208	1.263	1.414	2.025	2.036	2.162
2.211	2.370	2.532	2.693	2.805	2.910	2.912	3.192	3.263	3.348
3.348	3.427	3.499	3.534	3.767	3.751	3.858	3.986	4.049	4.244
4.323	4.381	4.392	4.397	4.647	4.753	4.929	4.973	5.074	4.381

**Table 6:** 

0.030	0.493	0.855	1.184	1.283	1.480	1.776	2.138	2.500	2.763
2.993	3.224	3.421	4.178	+4.441	5.691	+5.855	6.941	+6.941	+7.993
8.882	8.882	+9.145	11.480	11.513	+12.105	12.796	+12.993	+13.849	+16.612
+17.138	20.066	+20.329	+22.368	+26.776	+28.717	+28.717	+32.928	+33.783	+34.221
+34.770	+39.539	+41.118	+45.033	+46.053	+46.941	+48.289	+57.401	+58.322	+60.625

**Table 7:** 

0.658	0.822	1.414	2.500	3.322	3.816	4.737	+4.836	4.934	5.033
5.757	5.855	5.987	6.151	6.217	+6.447	8.651	8.717	+9.441	10.329
11.480	12.007	+12.007	12.237	+12.401	+13.059	+14.474	+15.000	15.461	15.757
16.480	16.711	+17.204	17.237	+17.303	+17.664	18.092	+18.092	+18.750	+20.625
23.158	+27.730	+31.184	+32.434	+35.921	+42.237	+44.638	+46.480	+47.467	+48.322
56.086									

**Table 8:** 

10	14	14	14	14	14	15	17	18	20
20	20	20	20	23	23	24	26	30	30
+30	+30	+30	+30	+30	31	40	49	51	52
60	+60	61	67	71	74	75	87	96	105
107	107	107	116	150	+150	+150	+150	+150	+150
+185									

**Table 9:** 

+0.14	+0.14	+0.29	+0.43	0.43	+0.57	+0.57	+1.86	2.86	+3.00
+3.00	3.14	3.14	+3.29	+3.29	3.43	3.43	3.71	3.86	+6.00
+6.00	+6.14	6.14	6.86	+8.71	9.00	9.43	+10.57	10.71	10.86
11.14	+11.86	13.00	14.43	+15.57	15.71	+16.57	+16.57	+17.29	18.43
18.57	+18.71	20.71	+21.29	23.86	+26.00	+27.57	29.14	29.71	+32.14
+33.14	40.57	48.57	49.43	53.86	61.86	66.57	68.71	68.96	72.86
72.86									

## Data Availability

The data was mentioned along the paper.

## Notations

IFR: Increasing failure rate
IFRA: Increasing failure rate average
NBU: New better than used
NB(W)UC: New better (worse) than used in a convex ordering
NBRUL: New better than renewal used in Laplace transform ordering
NBRU_mgf_: New better than renewal used in moment generating function
NBU_mgf_: New better than used in moment generating function
NBUCL: New better (worse) than used in a convex Laplace ordering
UBA: Used better than age
UBAC: Used better than age in convex order
UBAC (2): Used better than age in concave order
UBAL: Used better than age in Laplace transform
UBA_mgf_: Used better than age in moment generating function
RNBU_mgf_: Renewal new better than used in moment generating function.
